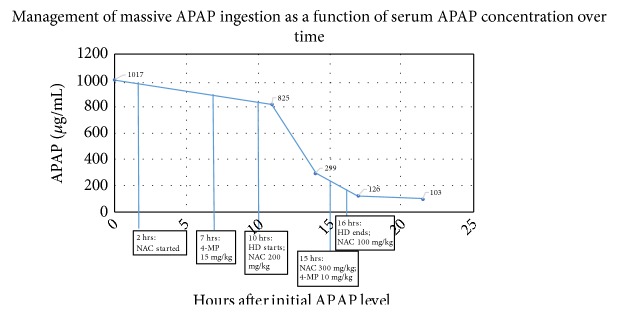# A Case Report of Massive Acetaminophen Poisoning Treated with a Novel “Triple Therapy”: N-Acetylcysteine, 4-Methylpyrazole, and Hemodialysis

**DOI:** 10.1155/2019/9301432

**Published:** 2019-03-05

**Authors:** Emily A. Kiernan, Julie A. Fritzges, Kathryn A. Henry, Kenneth D. Katz

**Affiliations:** ^1^Department of Emergency and Hospital Medicine, Lehigh Valley Health Network and University of South Florida Morsani College of Medicine, Lehigh Valley Campus, Cedar Crest Boulevard & I-78, Allentown, PA 18103, USA; ^2^University of South Florida Morsani College of Medicine, 12901 Bruce B. Downs Boulevard, Tampa, FL 33612, USA

## Abstract

Massive acetaminophen (N-acetyl-p-aminophenol; APAP) ingestion is characterized by a rapid onset of mitochondrial dysfunction, including metabolic acidosis, lactemia, and altered mental status without hepatotoxicity which may not respond to the standard doses of N-acetylcysteine (NAC). A 64-year-old woman without medical history presented comatose after an ingestion of 208 tablets of Tylenol PM™ (APAP 500 mg and diphenhydramine 25 mg). The initial APAP concentration measured 1,017 *µ*g/mL (therapeutic range 10-30 *µ*g/mL), and elevated anion gap metabolic acidosis, lactemia, and 5-oxoprolinemia were detected. High-dose intravenous (IV) NAC, 4-methylpyrazole (4-MP), and hemodialysis (HD) were initiated. She was transferred to a liver transplant center and continued both NAC and HD therapies until complete resolution of metabolic acidosis and coma without developing hepatitis. She was discharged without sequelae. This is the fourth highest APAP concentration recorded in a surviving patient. Moreover, this is the first report of a novel “triple therapy” using NAC, 4-MP, and HD in the setting of massive APAP ingestion that presents with coma, elevated anion gap metabolic acidosis, and lactemia. Emergency physicians should recognize these critically ill patients and consider high-dose NAC, 4-MP, and HD to be initiated in the emergency department (ED).

## 1. Introduction

In the United States, APAP toxicity is the most common cause of acute hepatic failure and is the second most common cause of liver failure requiring transplantation. The Annual Report of the American Association Poison Control Centers' National Poison Data System documented 49,417 cases of APAP exposure in 2016 and 21,776 cases of APAP combination medication exposure. APAP alone and APAP combinations resulted in 92 and 42 deaths, respectively [1].

Massive APAP ingestion is characterized by rapid onset of mitochondrial dysfunction, including metabolic acidosis, lactemia, and altered mental status without hepatotoxicity [2]. Metabolic acidosis in the setting of APAP poisoning is typically a late-onset process secondary to liver failure. However, when metabolic acidosis is present early after ingestion, it may be secondary to overwhelming N-acetyl-p-benzoquinone imine (NAPQI) presence. APAP can also cause 5-oxoprolinemia associated with depletion of liver glutathione stores and may contribute to metabolic acidosis early in clinical presentation, unassociated with liver failure [3, 4]. This is typically present when glutathione stores are exhausted, most often in those with genetic glutathione disorders and chronic APAP ingestion, but it has rarely been associated with acute toxicity [4, 5].

This case report details the case of a woman who intentionally ingested a massive amount of APAP and demonstrated both mitochondrial dysfunction and 5-oxoprolinemia contributing to metabolic acidosis, lactemia, and altered mental status. The authors of this paper suggest a novel approach to massive APAP toxicity through concomitant use of high-dose, IV NAC, 4-MP, and HD. This case represented the fourth highest APAP concentration in the literature of a surviving patient, as well as the first known report of administering 4-MP—a CYP450 2E1 inhibitor—in the successful management [6].

## 2. Case Presentation

A 64-year-old woman with no past medical history and no prescribed medications was found unresponsive at home after ingestion of 208 tablets of Tylenol PM™ (APAP 500 mg with diphenhydramine 25 mg) approximately three hours prior to transportation to the ED. She was endotracheally intubated by prehospital staff due to decreased level of consciousness, vomitus, and agonal respiration. On arrival to the ED, vital signs included temperature of 33.9°C, heart rate of 57 bpm, blood pressure of 139/102 mmHg, respiratory rate of 19 rpm, and oxygen saturation of 99% on 100%* Fi*O_2_. Physical examination demonstrated a Glasgow Coma Scale score 3 without spontaneous respiration. The patient subsequently became hypotensive requiring five IV push-dose epinephrine doses (total 100 *µ*g), followed by dopamine (10 *µ*g/kg/min increased to 15 *µ*g/kg/min), and then a norepinephrine infusion (10 *µ*g/min). An electrocardiogram (ECG) showed sinus rhythm of 58 bpm, PR of 144 ms, QRS of 112 ms, and QTc of 659 ms. Serum chemistries measured as follows: lactate 7.6 mmol/L (0.5-1.0 mmol/L), glucose 193 mg/dL (70-100 mg/dL), Na^+^ 142 mEq/L (136-144 mEq/L), K^+^ 3.2 mEq/L (3.7-5.2 mEq/L), Cl^−^ 110 mmol/L (96-106 mmol/L), CO_2_ 18 mmol/L (20-29 mmol/L), BUN 16 mg/dL (7-20 mg/dL), Cr 1.17 mg/dL (0.6-1.1 mg/dL), AST 21 IU/L (10-34 IU/L), ALT 99 IU/L (8-37 IU/L), and INR 1.2 (0.8-1.1). Initial ABG measured as follows: pH 7.32 (7.35-7.45), pCO_2_ 30 mmHg (35-45 mmHg), pO_2_ 249 mmHg (80-100 mmHg), and HCO_3_ 16 (CMV* Fi*O_2_ 65%, PEEP 6, RR 16, and TV 500 mL). Initial serum APAP concentration measured 1,017 *µ*g/mL. Serum salicylate and ethanol concentrations measured 7 mg/dL and negative, respectively. A preliminary urine drug screen of abuse detected only methadone. Expanded serum liquid chromatography/mass spectroscopy detected caffeine, dihydrocodeine/hydrocodol, lidocaine, monoethylglycinexylidide, and diphenhydramine. A chest X-ray revealed mild interstitial edema. Head computed tomography was unremarkable. The patient was admitted to the intensive care unit. There was no repeat ECG performed prior to transfer to transplant center; however, no dysrhythmias were observed on telemetry monitoring.

**Figure 1:**
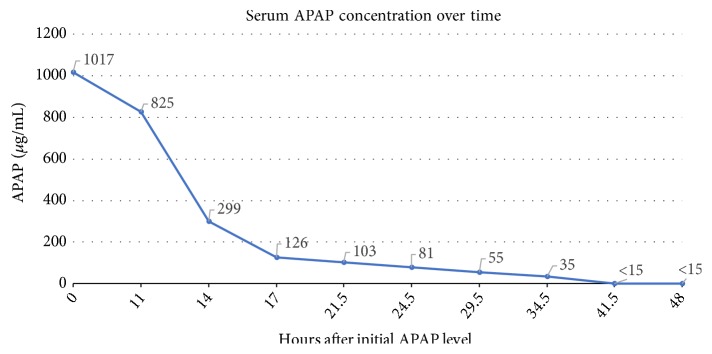


The medical toxicology service was consulted and recommended IV sodium bicarbonate (for prolonged QRS interval), IV NAC, IV 4-MP (15 mg/kg), and immediate HD. APAP concentration decreased to 825 *µ*g/mL after initiation of IV NAC, and serial concentrations exponentially decreased during “triple therapy” (Figures 1 and 2). During HD, the IV NAC rate of administration was doubled to 200 mg/kg and then tripled to 300 mg/kg, and a subsequent dose of IV 4-MP 10 mg/kg was administered; these were performed due to concern of HD removal of both antidotes.

The patient's mental status improved during HD; however, she did not follow commands. Due to concern for potential severe liver injury given the massive initial APAP concentration and lack of institutional transplant services, she was transferred to a liver transplant center on hospital day one. On arrival to the liver transplant center, she was maintained on IV NAC and received an additional HD treatment. 4-MP was not readministered. IV NAC was discontinued when APAP concentrations were undetectable. The patient was awake and following commands but failed extubation due to respiratory distress and pneumonia. She was ultimately extubated to BiPAP and discharged to an inpatient psychiatry unit approximately eight days after ED presentation in a normal state of health.

## 3. Discussion

Massive APAP ingestion results in saturation of hepatic sulfation and glucuronidation which leads to excessive metabolism via hepatic CYP450 2E1 to the toxic metabolite, NAPQI. NAPQI inhibits mitochondrial respiration and contributes to cellular toxicity and metabolic acidosis [2]. Accumulation of the organic acid, 5-oxoproline, may also contribute to the metabolic acidosis. Although typically caused by genetic glutathione deficiencies, by chronic APAP use, or with coexisting conditions such as sepsis, malnutrition, or pregnancy, 5-oxoprolinemia may be present in acute, massive APAP ingestion alone [4, 5]. 5-Oxoproline is an intermediate in the gamma-glutamyl pathway, responsible for regenerating glutathione and for transporting amino acid into the cytosol. Normal glutathione concentrations are required for negative feedback on the enzyme gamma-glutamyl cysteine synthase. When glutathione stores are depleted, there is overproduction of gamma-glutamyl cysteine, which is partially metabolized to 5-oxoproline, ultimately leading to acidosis. In this patient, given her coexisting 5-oxoprolinemia, it is logical that both NAPQI and organic acid accumulation contributed to her condition. The association with altered mental status after APAP ingestion with 5-oxoprolinemia has been reported [4, 5].

Standardly accepted treatment of APAP toxicity includes restoration of glutathione stores with NAC administration. However, given the considerable NAPQI formation in the setting of massive APAP ingestion, administration of a CYP450 2E1 substrate such as 4-MP or ethanol may lessen NAPQI formation and thus obviate significant cellular toxicity [7, 8]. 4-MP is a competitive antagonist of alcohol dehydrogenase and a potent CYP450 2E1 inhibitor that is used in the treatment of ethylene glycol and methanol toxicity. 4-MP has been studied as an alternative to NAC in rats with APAP-induced hepatotoxicity by Küçükardalı et al. The authors compared the efficacy of NAC and 4-MP alone and in combination, ultimately concluding that the two xenobiotics have similar efficacy in limiting hepatotoxicity, as reflected by lower levels of serum transaminases and lesser degrees of hepatic necrosis [9]. In addition, a recent study by Akakpo et al. investigated the protective effect of 4-MP alone in treatment of APAP-induced liver injury in both mice and primary human hepatocytes. When 4-MP 50 mg/kg was administered with APAP 300 mg/kg, the expected severe liver injury after six hours was almost completely eliminated. In addition, the use of 4-MP resulted in inhibiting the formation of APAP-glutathione conjugates, reducing the depletion of liver glutathione stores, and preventing APAP-induced human hepatocyte cell death. The 50 mg/kg 4-MP dose used in the mice by Akakpo et al. is approximately equivalent to 4 mg/kg in humans; theoretically, 15 mg/kg 4-MP is several times higher than the effective dose in mice and is also the approved dose for humans in toxic alcohol poisoning. However, a therapeutic dose of 4-MP in humans for APAP poisoning would need to be elucidated [10]. In the presence of 4-MP, NAPQI formation via CYP450 2E1 is reduced [11]. Two studies demonstrated that ethanol caused a variable and short-acting induction of CYP450 2E1 in humans, appearing to involve several mechanisms including the stabilization of the 2E1 protein ultimately leading to simultaneous inhibition and induction [12–14]. Furthermore, acute ethanol ingestion in the setting of an APAP overdose appears to be hepatoprotective in humans. The protective effect disappears when ethanol is eliminated [14, 15]. Given the potential adverse, dose-related effects of ethanol administration—first-order clearance, intoxication, and hypoglycemia—4-MP administration would be a preferable substrate. The benefits include simple dosing regimen, no need to monitor serum concentrations, a wider therapeutic index, longer duration of action, and more predictable kinetics [16, 17].

APAP is amendable to elimination via HD due to its molecular weight of 151 Da, V_d_ of 1 L/kg, and total protein binding of 10-30%. In addition, use of HD rapidly improves both severe metabolic derangement and APAP clearance [18]. Ghannoum et al. reported that HD doubles APAP clearance, and APAP half-life decreased from 5.2 hours to 1.9 hours. Clearance of NAC is also increased with HD and, thus, the recommended infusion rate is increased two to three times while the patient is on HD [2, 17, 19].

## 4. Conclusion

A novel “triple therapy” with NAC, 4-MP, and HD may be applicable and have physiologic merit in the case of a patient suffering from massive APAP poisoning. Further study is warranted.

## Figures and Tables

**Figure 2:**